# A Treatable Genetic Disease Caused by CAD Mutation

**DOI:** 10.3389/fped.2022.771374

**Published:** 2022-03-09

**Authors:** Xia Peng, Li-ping Xia, Hai-ju Zhang, Jing Zhang, Shi-qian Yu, Shun Wang, Yu-ming Xu, Baozhen Yao, Jingping Ye

**Affiliations:** Department of Pediatrics, Renmin Hospital of Wuhan University, Wuhan, China

**Keywords:** early infantile epileptic encephalopathies, CAD, treatment, uridine, genetic disease

## Abstract

Type 50 early infantile epileptic encephalopathy, or EIEE-50 for short, is an autosomal recessive genetic disorder resulting from CAD mutations. So far, little has been reported on the disease. In this article, we will discuss the case of a male infant who is 8 years and 5 months old. A whole-exome sequencing of the boy revealed CAD compound heterozygous mutations. He suffered from global developmental delay and regression, refractory epilepsy, and anemia. After his diagnosis, we used uridine treatment and gained encouraging results. In this article, we will analyze our case studies in the context of the literature, so as to improve pediatricians' understanding of the disease.

## Introduction

With the continuous development of precision medicine, we can reach the stage of molecular diagnosis of disease. Sadly, however, progress in identifying the molecular basis has not always translated into effective treatments (except in a few cases including EIEE-50). EIEE-50 is an autosomal invisible genetic disorder caused by a CAD mutation that encodes a multifunctional enzyme complex involved in *de novo* pyrimidine biosynthesis (1). When a CAD gene mutates, it will cause a nascent barrier to pyrimidine synthesis. In normal cells, the activation of the *de novo* pyrimidine synthesis pathway is essential for satisfying the nucleotides required for DNA and RNA replication. When this pathway is destroyed, not only will the biosynthesis of pyrimidine be impaired, but the level of glycosylated precursor will also be reduced, which will seriously affect the normal proliferation and metabolism of cells (2).

The clinical manifestations of EIEE-50 can be categorized into three main signs: global growth retardation or degeneration, refractory epilepsy, and anemia with anisopoikilocytosis (3, 4). In addition, some patients may suffer from optic nerve damage (5). This disease can be cured at an early stage with oral uridine. Otherwise, if left untreated, the disease is usually fatal.

## Case Report

An 8-year-plus-5-month-old boy was hospitalized with refractory epilepsy. He was born at full term without perinatal abnormalities. His parents and little sisters were healthy, and the parents did not marry close relatives. Unluckily, the boy had slow motor function development. It was not until 12 months before he could sit alone and 30 months before he could walk. Later, when he was 3 months old, it was discovered that he had anemia and his hemoglobin levels fluctuated between 69 and 110 g/L without a definite diagnosis. He was treated with red blood cell transfusions and immunoglobulin. After 9 months, he underwent surgery for left hydronephrosis.

The seizures began with a 2-year-old febrile seizure. When he was 7 years and 4 months old, the seizures gradually developed into afebrile seizures, namely, generalized tonic–clonic convulsion lasting 1–2 min and occurring two-to-three times a day. Two months later, he was treated with an antiepileptic drug (valproic acid). After relapse, the level of valproic acid increased to 25 mg/kg/day. However, there was no significant remission, so another antiepileptic drug was added (perampanel). When he was 8 years and 4 months old, he tried two antiepileptic drugs, but the frequency of epileptic seizures did not decrease—up to 20 attacks a month. Meanwhile, his cognitive and motor functions were under observation. He could not eat, sit, stand alone, or communicate with others, and he had hypersomnia. As a result, he had to accept further examination.

**Figure 1:**
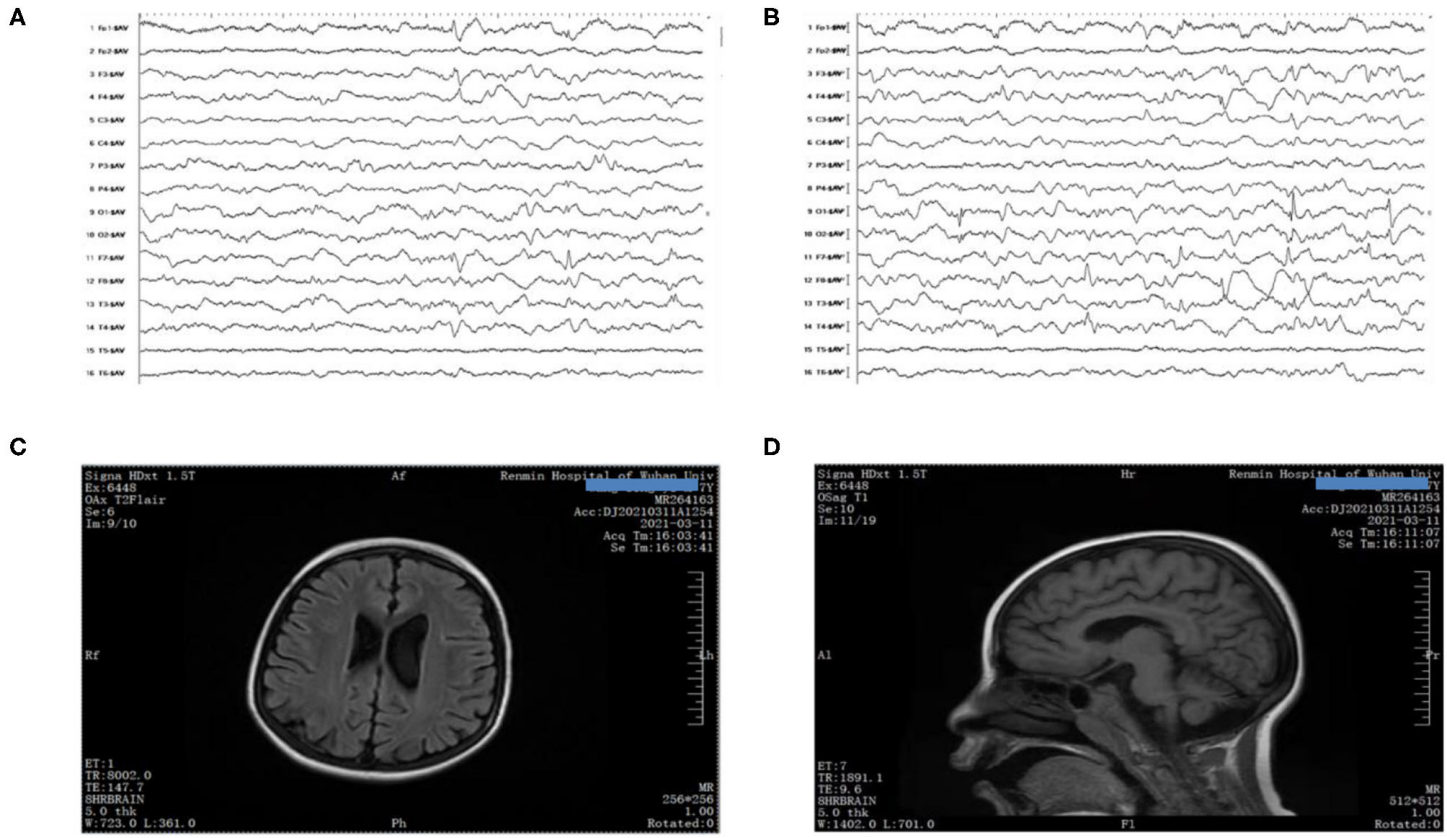
**(A,B)** EEG showed poor background with multiple spikes and spike-and-wave discharges. **(C,D)** Brain MRI presented mega cisterna magna, together with enlarged left lateral ventricle.

When the boy was 8 years and 4 months old, he began to suffer from electroencephalogram (EEG) with multiple spikes and spike-and-wave discharges, and persistent low voltages were also detected at some electrodes (Figures 1A,B). Due to developmental delay and corpus callosum aplasia, his first magnetic resonance imaging (MRI) was taken when he was 10 months old. Therefore, a combination of mNGF (mouse nerve growth factor) and rehabilitation was used. mNGF was extracted from the mouse submandibular gland, which could promote the survival, growth, differentiation, and regeneration of peripheral nerve and central neuron (6). An MRI examination 7 months later revealed mild enlargement of the left lateral ventricle and abnormal signal in the bilateral ventricles, suggesting a delayed formation of the myelin sheath in the periventricular white matter. Also, when the boy was 8 years and 4 months old, the MRI showed a large cisterna occipitalis with an enlargement of the left lateral ventricle (Figures 1C,D). Routine blood tests indicated the anemia that persisted during his hospitalization.

**Figure 2:**
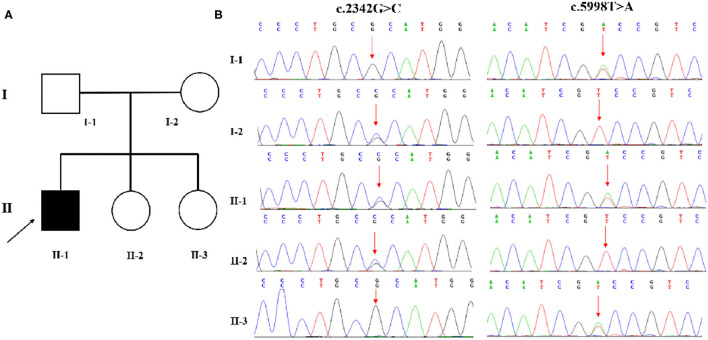
**(A,B)** WES identified two variations in CAD. A missense (c.2342G>C: p.R781P) variant was inherited from the proband's mother, and the other (c.5998T>A: p.S2000T) variant came from his father.

Family-based whole-exome sequencing (WES) and Sanger sequencing were given to the boy and all his family members; compound heterozygous variants in the CAD gene were identified in the proband. A missense (c.2342G>C: p.R781P) variant came from the proband's mother, and the other missense (c.5998T>A: p.S2000T) variant came from the proband's father (Figures 2A,B). According to the guidelines of the American College of Medical Genetics and Genomics and the Association for Molecular Pathology, both variations were interpreted as potentially pathogenic variants associated with EEIE-50.

During hospitalization, the boy received nasogastric tube feeding, red blood cell transfusion, intravenous potassium, and other supportive therapy. Furthermore, he was treated with an oral uridine dose of 100 mg/kg/day. After jabbing oral uridine, the boy was surprisingly better; he was able to sit alone for a few seconds, his mental status improved, and no more seizures were observed. Things were not always good; he was still unable to eat, walk alone, or communicate with others. A follow-up EEG examination after uridine treatment was rejected.

## Discussion

EIEE is a refractory form of epilepsy that begins in infancy and is thought to be associated with a variety of causes, including hereditary, metabolic disorders, brain abnormalities, and brain damage. So far, nearly 100 genes related to EIEE have been identified, which can be roughly divided into ion channel genes, neurotransmitter receptor genes, and other genes related to cell metabolism and signal transduction. Most of these diseases cannot be cured with several exceptions. Recently, a new treatable category of EIEE has attracted public attention, namely, EIEE-50, caused by mutations in the CAD gene (7). Without timely treatment, this disease is progressive and fatal. However, a growing number of reports claim that there are several patients who have successfully relieved their symptoms thanks to oral uridine (3, 4).

**Figure 3:**
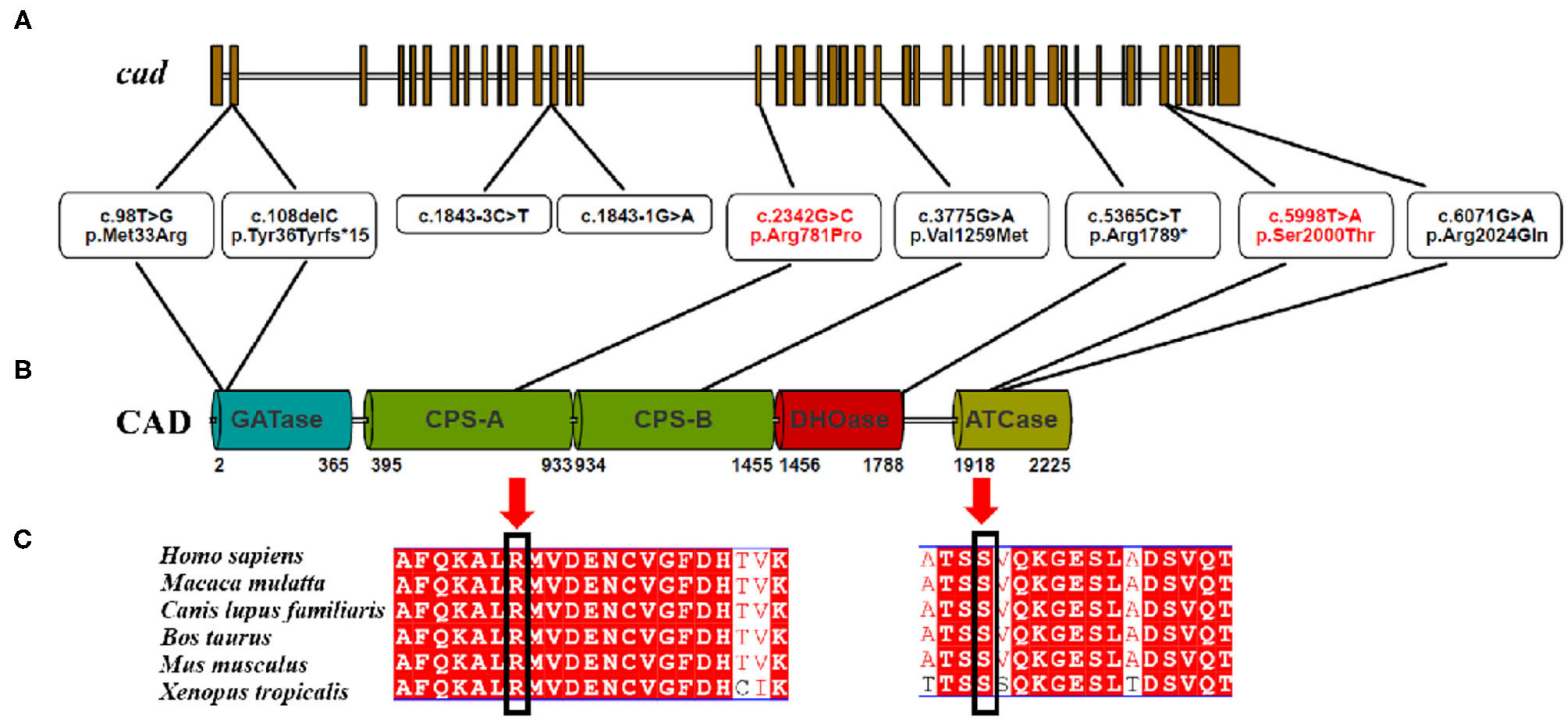
**(A–C)** CAD is evolutionally conserved among different species.

CAD, which is located on chromosome 2 (2p23.3), encodes a highly conserved multifunctional enzyme complex related to the synthesis of *de novo* pyrimidine. This complex consists of three parts, including carbamoyl-phosphate synthetase 2, aspartate transcarbamylase, and dihydroorotase (1) (Figure 3). The more conserved the amino acids are in the evolution of the species, the more important amino acid sites are for maintaining protein function. A comparison between the multi-sequence alignment of CAD and amino acid sequences revealed that amino acid site 129 was conserved in these species, including *Homo sapiens, Macaca mulatta, Canis lupus familiaris, Bos taurus, Mus musculus*, and *Xenopus tropicalis*. Mutations in conserved proteins make them vulnerable to disease.

EIEE-50 is a newly discovered disease in recent years since 2015, when Ng et al. reported the first one. In that case, the patient was diagnosed with pandisylase deficiency at 6 months and with renal tubular acidosis at 1 year. When he was 1 year and 5 months old, he had seizures and fine motor and language problems, and laboratory auxiliary tests suggested that he had anemia with anisopoikilocytosis. Finally, at the age of 4, he was admitted to the National Institute of Health (NIH) Medical Center and diagnosed with EIEE-50 by WES (8). In 2017, four children with EIEE-50 from three different families were introduced by Koch and his colleagues. Of these four patients, two were siblings and their parents were close relatives. The four children showed similar clinical features, including global developmental delay or regression, refractory epilepsy, and anemia with ischemic polycythemia. In addition, one of them had strabismus, but the Koch study did not directly connect strabismus to EIEE-50 (3). In 2019, a team led by Zhou presented the first Chinese patient with EIEE-50, whose clinical manifestations were consistent with previous studies. Later, this team reported another two cases of EIEE-50 in mainland China. On the basis of confirming the original clinical findings, they found that the children with EIEE-50 also had optic nerve damage caused by optic nerve transmission block. This finding was consistent with Koch's observations and had improved the clinical features of EIEE-50 (4, 5). Subsequent reports on the clinical manifestations of this disease were coherent with previous studies (9). In our case, the child's clinical features were in line with those previously published. The child endured anemia when he was 3 months old and febrile convulsion when he was 2 and gradually suffered from non-febrile convulsion that could not be well controlled by two antiepileptic drugs. The child had a history of backwardness. Prior to admission, he showed significant developmental regression. In our case, however, we did not find any visual impairment in the child, nor did we do any tests to confirm whether the child had optic nerve damage.

EEG, brain MRI, and blood routine examinations are the auxiliary examinations of the essence of EIEE-50. They have some regularity, but they are far from enough to locate EIEE-50. Previous research found that an abnormal background rhythm with epileptic-like discharge was a common feature of EEG (3, 10). In our case, the situation was the same. Our patient showed persistent low voltage on some electrodes. Beyond that, multifocal spikes and spike-and-wave were also detected. Based on our understanding, such a kind of EEG reflects the state of epileptic encephalopathy, which is not the unique feature of EIEE-50. As for craniocerebral MRI, it could be normal in the early stages. With the development of the disease, many cases appeared with global brain atrophy, suggesting that the lesions in the brain may be continuous. Although previous articles have shown that brain atrophy occurs in children as early as 3.5 years of age, our case did not show the sign. A routine blood examination ordinarily shows moderate anemia with anisopoikilocytosis. In our report, anemia was the predominant symptom in this child.

The above-mentioned means are important detecting procedures, though the specificity is not enough. This reminds us that we need to consider the possibility of EIEE-50 combined with the results of auxiliary examination in clinical cases of children with refractory epilepsy, developmental delay, and anemia. Undoubtedly, the definite diagnosis of this disease depends on WES.

CAD mutation is the pathogenesis of this disease, and the main function of CAD is to participate in the biological synthesis of pyrimidine, serving as compensatory substances to promote the formation of pyrimidine in a therapeutic approach. Uridine can be ingested and phosphorylated by cells, which provides a mechanism to overcome the defects of pyrimidine nucleoside, hence serving as an irreplaceable drug for the treatment of EIEE-50. On the basis of the information available, uridine has been successfully applied to deal with EIEE-50. Uridine was first used for EIEE-50 in 2015, in which case the child was treated with oral uridine but the results were not described in detail (1). It was not until 2017 that Koch and his teammates elaborated on the effects of uridine on EIEE-50. Of the four children according to his article, two died at 4 and 5, respectively, because they did not receive timely treatment. The other two who received oral uridine had their seizures stopped, and their conditions improved significantly (3). Since 2019, Chinese researchers have begun to recognize and treat EIEE-50, as favored by the following results (4, 5). Since then, clinicians around the globe have become more aware of EIEE-50 and have increased the use of uridine. In our case, uridine was given at a dose of 100 mg/kg immediately after diagnosis, and no significant side effects were observed. A week later, the seizures stopped, and the boy made more eye contact with others. He could sit alone for a few seconds, though he could not stand, eat alone, or talk to others. After a month of taking oral uridine, his mother told us that he could eat and sit alone but still could not walk alone.

## Conclusion

EIEE-50, caused by a CAD mutation, will lead to intractable epilepsy, developmental delay or regression, and anemia in children. Some patients may also suffer from impaired optic nerves. Normally, EEG shows the change of background rhythm and epileptiform discharge. Early craniocerebral MRI may show normal or mild abnormalities. As the disease progresses, brain atrophy may occur. Uridine (with a dosage of 100 mg/kg/day) is an effective drug for the treatment. The children who were treated with uridine clearly improved, and the remission of symptoms was closely related to the course of the disease. Genetic testing is a tool to confirm this disease. EIEE-50 is one of the few genetic diseases that can be treated, and the results are favorable, highlighting the importance and value of precision medicine.

## Data Availability Statement

The original contributions presented in the study are included in the article/supplementary material, further inquiries can be directed to the corresponding author.

## Ethics Statement

Written informed consent was obtained from the individual(s) for the publication of any potentially identifiable images or data included in this article.

## Author Contributions

XP and BY drafted the article. L-pX and H-jZ helped in correcting the mistakes. XP, JZ, S-qY, SW, Y-mX, and BY were responsible for collecting the dates. JY did great jobs in the revision process. All authors contributed to the article and approved the submitted version.

## Funding

This study was supported by Medical Science Advancement Program of Wuhan University (No. TFLC2018001).

## Conflict of Interest

The authors declare that the research was conducted in the absence of any commercial or financial relationships that could be construed as a potential conflict of interest.

## Publisher's Note

All claims expressed in this article are solely those of the authors and do not necessarily represent those of their affiliated organizations, or those of the publisher, the editors and the reviewers. Any product that may be evaluated in this article, or claim that may be made by its manufacturer, is not guaranteed or endorsed by the publisher.

## References

[1] Chen KC Vannais DB Jones C Patterson D Davidson JN. Mapping of the gene encoding the multifunctional protein carrying out the first three steps of pyrimidine biosynthesis to human chromosome 2. Hum Genet. (1989) 82:40–4. 10.1007/BF00288269 2565865

[2] Shi X. Prognostic evaluation of CAD gene in pancancer. Tumor Found Clin. (2020) 33:485–91.

[3] Koch J Mayr JA Alhaddad B Rauscher C Bierau J Kovacs-Nagy R. CAD mutations and uridine-responsive epileptic encephalopathy. Brain. (2017) 140:279–86. 10.1093/brain/aww300 28007989

[4] Zhou L Xu H Wang T Wu Y. A patient with CAD deficiency responsive to uridine and literature review. Front Neurol. (2020) 11:64. 10.3389/fneur.2020.00064 32117025 PMC7012989

[5] Zhou L Deng J Stenton SL Zhou J Li H Chen C. Case report: rapid treatment of uridine-responsive epileptic encephalopathy caused by CAD deficiency. Front Pharmacol. (2020) 11:1956. 10.3389/fphar.2020.608737 33364968 PMC7750521

[6] Yuan JJ Wang WW Duan J Xu XY Tang JL. A prospective randomized controlled study on mouse nerve growth factor in the treatment of global developmental delay in children. Chin J Contemp Pediatr. (2021) 23:786–90. 34511166 10.7499/j.issn.1008-8830.2106042 PMC8428910

[7] Li Y Wang B. Advances in molecular genetics of early infantile epileptic encephalopathy. Electron J Dev Med. (2018) 6:58–64.

[8] Ng BG Wolfe LA Ichikawa M Markello T He M Tifft CJ. Biallelic mutations in CAD, impair de novo pyrimidine biosynthesis and decrease glycosylation precursors. Hum Mol Genet. (2015) 24:3050–7. 10.1093/hmg/ddv057 25678555 PMC4424951

[9] McGraw CM Mahida S Jayakar P Koh HY Taylor A Resnick T. Uridine-responsive epileptic encephalopathy due to inherited variations in CAD: a tale of two siblings. Ann Clin Transl Neurol. (2021) 8:716–22. 10.1002/acn3.51272 33497533 PMC7951104

[10] Kamate M Patil S. CAD deficiency-another treatable early infantile epileptic encephalopathy. Pediatr Neurol. (2020) 110:97–8. 10.1016/j.pediatrneurol.2020.05.001 32654958

